# Nanostructured giant magneto-impedance multilayers deposited onto flexible substrates for low pressure sensing

**DOI:** 10.1186/1556-276X-7-230

**Published:** 2012-04-23

**Authors:** Eduardo Fernández, Galina V Kurlyandskaya, Alfredo García-Arribas, Andrey V Svalov

**Affiliations:** 1 Departamento de Electricidad y Electrónica, Universidad del País Vasco UPV/EHU, Bilbao, 48080, Spain

**Keywords:** giant magneto-impedance, thin film, multilayer, magnetic sensor, pressure sensor

## Abstract

Nanostructured FeNi-based multilayers are very suitable for use as magnetic sensors using the giant magneto-impedance effect. New fields of application can be opened with these materials deposited onto flexible substrates. In this work, we compare the performance of samples prepared onto a rigid glass substrate and onto a cyclo olefin copolymer flexible one. Although a significant reduction of the field sensitivity is found due to the increased effect of the stresses generated during preparation, the results are still satisfactory for use as magnetic field sensors in special applications. Moreover, we take advantage of the flexible nature of the substrate to evaluate the pressure dependence of the giant magneto-impedance effect. Sensitivities up to 1 Ω/Pa are found for pressures in the range of 0 to 1 Pa, demostrating the suitability of these nanostructured materials deposited onto flexible substrates to build sensitive pressure sensors.

## Introduction

**Figure 1:**
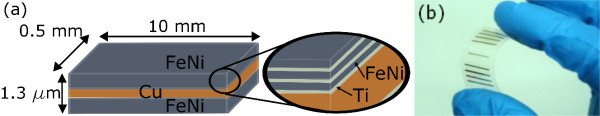
**Scheme of the GMI structure and the samples deposited onto a COC substrate**. (**a**) Scheme of the [FeNi(170 nm)/Ti(6 nm)]_3_/Cu(250 nm)/[Ti(6 nm)/FeNi(170 nm)]_3 _GMI structure used in this study. (**b**) Samples deposited onto a COC flexible substrate.

The giant magneto-impedance (GMI) effect is the great change of the electrical impedance that soft ferromagnetic materials exhibit when a magnetic field is applied. This effect is basically a consequence of the reduction of the material's effective cross-section when an AC current flows due to the skin effect. The impedance change can be described using Maxwell equations for specific boundary conditions [1,2]. It is controlled by the changes of the material's permeability caused by the application of an external magnetic field [3]. Thus, GMI applications require soft magnetic materials with low coercivity, very high magnetic permeability, high saturation magnetization and a well-defined magnetic anisotropy (*H_k_*). Amorphous Co-based wires and ribbons are the most studied GMI materials [4], but for sensor applications, thin film is a much more convenient geometry. One of the most suitable materials for this purpose is the Fe_19_Ni_81 _permalloy. For good GMI response, the magnetic film has to be about one micron thick. Previous studies [5] have demonstrated that, for FeNi films, once the thickness reaches 200 nm, an out-of-plane magnetization component appears, ruining the in-plane anisotropy and the magnetic softness required for high GMI values. The insertion of 6-nm thick titanium layers between successive 170-nm thick permalloy layers interrupts the developing of the out-of-plane component of the magnetization, allowing to obtain about one micron-thick films without deteriorating the magnetic softness [6]. It is also possible to increase the GMI performance even at lower frequencies, creating a sandwich structure by inserting a nonmagnetic layer between two magnetic stacks. This configuration enhances the magneto-inductive effect and allows to obtain a higher GMI ratio when the conductivities of both types of layers are different enough [7]. The structure of the material that we have used for this work is, therefore, a F/C/F sandwich, where F is the magnetic structure made of three successive combinations of 170 nm permalloy and 6 nm Ti layers, and C is a conductive, nonmagnetic 250-nm copper layer. This structure is depicted in Figure 1a.

## Experimental details

The GMI material used in this investigation is obtained by sputtering deposition from permalloy (Fe_20_Ni_80_), titanium and copper targets onto both glass and cyclo olefin copolymer (COC) substrates. The samples are deposited in the form of elongated strips 0.5 mm wide and 10 mm long using metallic masks (Figure 1b) under a magnetic field of 20 kA/m applied in the plane of the film to induce a well-defined transverse magnetic anisotropy. The background pressure was 3 × 10 ^-7 ^mbar and the Ar pressure during the deposition was 3.8 × 10^-3 ^mbar. The power used for the FeNi target was 100 W with a deposition speed of *V*_FeNi _= 26 nm/min and 60 W for titanium and cooper targets with a deposition speed of *V*_Ti _= 4 nm/min and *V*_Cu _= 25 nm/min.

COC is a transparent and flexible polymer that is used, for example, to fabricate micro-fluidic systems. Recently, a sensor to measure the magnetic-particle concentration in continuous flow has been proposed based in a COC micro-chamber with a GMI magnetic sensor underneath [8]. An obvious upgrade of such a system will be to deposit the sensing GMI material directly onto the COC material, as described in this work. To magnetically characterize the prepared samples, the hysteresis loops along the sample length were measured by vibrating sample magnetometry (VSM). The impedance measurements were performed using radio frequency (RF) techniques by gluing the sample with a conductive silver paint between two micro-strip lines with 50 Ω of characteristic impedance. The impedance was deduced from the scattering parameter *S*_11 _measured by a network analyzer using an RF input power of 0 dB (that corresponds to an excitation of about 1 mA across the sample) after proper calibration and mathematical subtraction of the test fixture contributions. Details of the measuring procedure can be found elsewhere [9]. The test fixture containing the sample is placed inside a pair of Helmholtz coils to provide the variable magnetic field. To study the performance of the samples deposited onto the COC substrates as pressure detectors, different weights were placed over a rectangular glass (14 × 18 mm) situated onto the sample, reaching a maximum pressure of 4 Pa. In this way, the sample's complex impedance *Z *was measured at different pressure values as a function of the external magnetic field in a frequency range of 300 kHz to 300 MHz. In this range, the ferromagnetic resonance effects are still unimportant, and quasi-static processes dominate the MI behavior [10]. The GMI ratio was defined with respect to the magnetically saturated sample in the maximum applied field of *H*_sat _= 12 kA/m. Also, GMI sensitivities $s\left(\frac{\Delta Z/Z}{\Delta H}\right)$were calculated by differentiating with respect to the magnetic field *H*.

## Results and discussion

**Figure 2:**
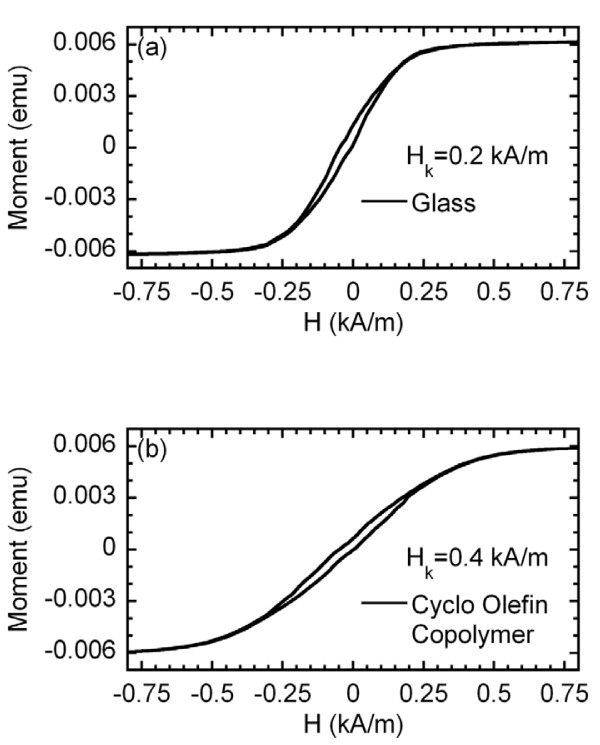
**M(H) curves**. (**a**) Sample deposited onto the rigid glass substrate and (**b**) onto the flexible substrate.

Figure 2 shows the VSM hysteresis loops for the samples deposited onto glass and COC, respectively, with the magnetic field applied in plane along the length of the sample and in the transverse direction with respect to the easy magnetization axis. The shape of both hysteresis loops confirms the formation of a rather well-defined, in-plane, transverse magnetic anisotropy. The sample deposited on COC displays a higher value of the anisotropy field (0.4 kA/m compared with 0.2 kA/m of the sample deposited onto glass), together with a larger dispersion of anisotropies, manifested by a more rounded approach to saturation. This behavior is probably caused by the stresses generated during the deposition process which are more important in the case of the flexible substrate.

**Figure 3:**
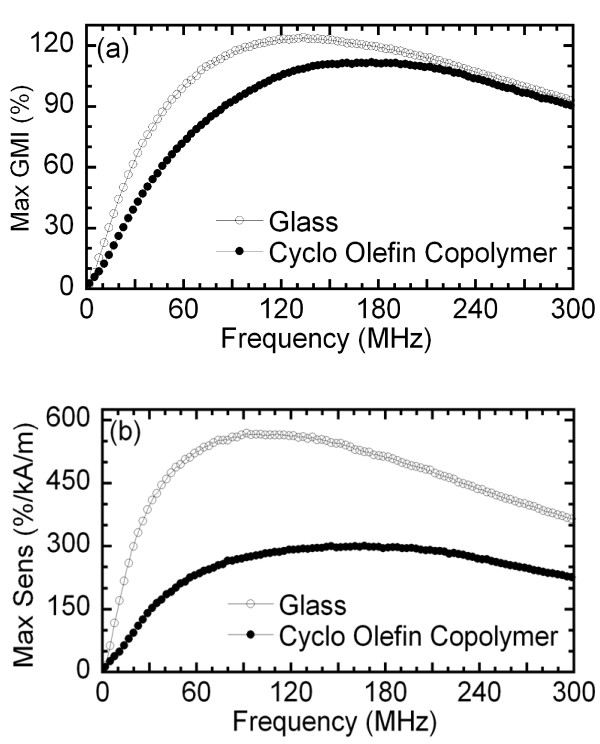
**Frequency dependence field sensitivity**. Frequency dependence of the maximum value of (**a**) the *Δ*Z/Z ratio and of (**b**) the field sensitivity.

An excellent GMI ratio is obtained for both samples (Figure 3a). For the one deposited onto glass, a 120% ratio of impedance change is found at 120 MHz, whereas for the sample deposited onto COC, the ratio is 110% at 180 MHz. However, the sensitivity to the magnetic field is greatly reduced in this last sample due to the increased value of the anisotropy field *H_k _*(Figure 3b). Even so, the sensitivity is still high (about 300%/kAm^-1^) due to the excellent performance of the multilayer nanostructure. These values can be probably increased using slightly different geometries, for instance, increasing the thickness of the nonmagnetic central layer [11] or by closing the transverse magnetic path by decreasing the width of the cooper central layer [12].

**Figure 4:**
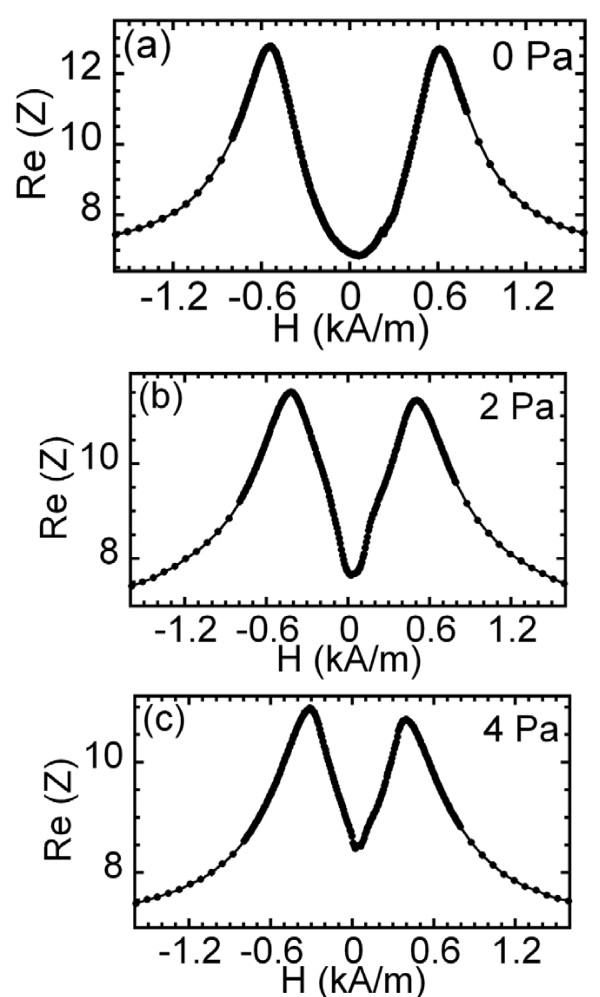
**Evolution of the GMI curves when subjected to increasing pressures**. Real part of the impedance as a function of the applied magnetic field at 100 MHz and with an applied pressure of (**a**) 0 Pa, (**b**) 2 Pa and (**c**) 4 Pa.

The flexible nature of the COC substrate allows to explore the possibility of developing a pressure sensor based on the changes of the GMI response. Figure 4 displays the evolution of the GMI curves (real part of the impedance as a function of the magnetic field measured at a frequency of 100 MHz) when subjected to increasing pressures. The most evident change takes place in the minimum value of the impedance at zero applied field and the distance between peaks and the values of the maxima change.

**Figure 5:**
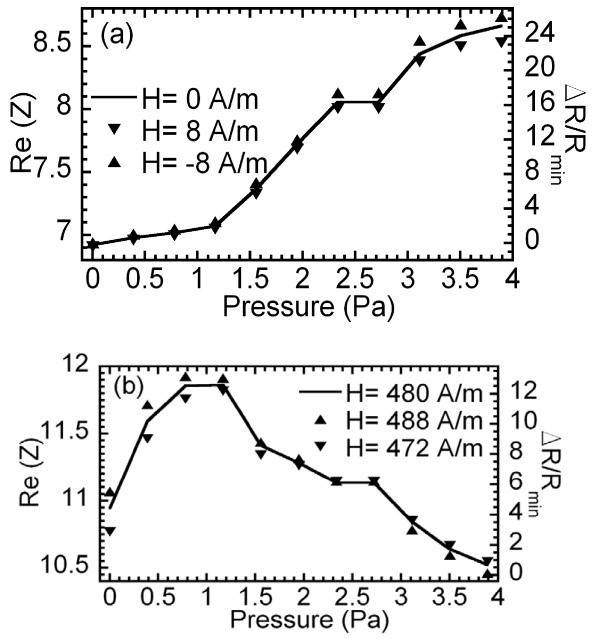
**Real part of the impedance variation**. Real part of the impedance as a function of the applied pressure at (**a**) *H *= 0 A/m and (**b**) *H *= 480 A/m, measured at 100 MHz.

Figure 5 quantifies these changes. The real part of the impedance at zero applied field (Figure 5a) monotonically increases with the applied pressure. A relative change of 25% is produced in this magnitude. The pressure dependence of the impedance at an applied field of 480 A/m, where the maximum field sensitivity at this frequency is reached, is also represented (Figure 5b). Although the behavior is not monotonous, a very steep change of impedance is observed at low pressures: 10% of change between 0 and 1 Pa.

In our experimental set-up, the applied magnetic field is continuously monitored, and its value is maintained stable using feedback control with an accuracy of 0.8 A/m. To demonstrate the stability of the pressure sensor against small changes in the applied field, both graphs (Figure 5a,b) display data corresponding to the impedance measured at different but close (8 A/m apart) values of the applied magnetic field. The results show that the impedance variation due to field fluctuations is very small (typically about 0.4%, 2% in the worst case). It is important to state that, for comparison, similar pressure measurements have been performed on the sample deposited onto glass. No observable pressure dependence has been found in this case.

## Conclusions

We have demonstrated first that excellent GMI response can be obtained from nanostructured multilayers deposited onto a flexible polymeric substrate. These magnetic nanostructures can be useful for a number of applications as detection of magnetic micro- and nanoparticles in micro-fluidic chambers that are fabricated using such materials [8]. On the other hand, we have studied the response of the sample deposited onto the flexible substrate to the applied pressure. The GMI curves display great changes as a function of pressure. At zero magnetic field, the impedance increases monotonically with an average slope of 0.4 Ω/Pa. When biased at the point of maximum field sensitivity, the sample presents a pressure sensitivity of about 1 Ω/Pa between 0 and 1 Pa. Focusing on possible pressure sensor applications, we have checked that the pressure dependence is quite stable when small magnetic field variations are present.

## Competing interests

The authors declare that they have no competing interests.

## Authors' contributions

All authors designed and developed this type of novel high frequency MI sensor. AVS and GVK did the study of the optimum conditions for nanostructures deposited onto flexible substrate and the magnetic characterization of the samples. AGA designed the GMI measurement setup, and EF performed the measurements and interpreted all the data. EF and AGA wrote the manuscript. All authors discussed the results and implications, and commented on the manuscript at all stages. All authors read and approved the final manuscript.

## Authors' information

Mr. Eduardo Fernández got his degree on physics at the University of the Basque Country in 2008. He is currently doing his PhD about magnetic field microsensors based on giant magnetoimpedance at the Department of Electricity and Electronics in the same university.

Dr. Galina V. Kurlyandskaya graduated from Ural State University, Ekaterinburg, Russia. She started her research work in 1983 at the Institute of Metal Physics UD RAS. She obtained her PhD in Physics of Magnetic Phenomena in 1990 and advanced Doctor of Science degree in 2007. Dr. Kurlyandskaya received an advanced training at the Institute of Applied Magnetism, University of Oviedo, University of the Basque Country, University of Dusseldorf named under Heinrich Heine, ENSCashan, University of Maryland and Ural State University A.M. Gorky. Her main research areas are fabrication, magnetic and transport properties of amorphous and nanostructured materials, magnetic domains, resonant and non-resonant magnetoabsorption, and magnetic sensors and biosensors.

Dr. Alfredo García-Arribas got his PhD degree at the Universidad del País Vasco (Spain) in 1996. He is currently with the Departamento de Electricidad y Electrónica in the same university. He is mainly interested in soft magnetic properties of materials and their application to magnetic sensors.

Dr. Andrey V. Svalov graduated from Ural State University, Ekaterinburg, Russia were he also obtained PhD in Physics of Magnetic Phenomenon in 2002. Dr. Svalov received advanced training at Ural State University, University of Oviedo and The Basque Country University UPV-EHU. For a long time, his main research activities were related to preparation and characterization of thin magnetic films and multilayers with special focus of Rare Earth containing nanostructures.
